# Epistasis confers resistance to Bt toxin Cry1Ac in the cotton bollworm

**DOI:** 10.1111/eva.12598

**Published:** 2018-02-10

**Authors:** Meijing Gao, Ximeng Wang, Yihua Yang, Bruce E. Tabashnik, Yidong Wu

**Affiliations:** ^1^ College of Plant Protection Nanjing Agricultural University Nanjing China; ^2^ Department of Entomology University of Arizona Tucson AZ USA

**Keywords:** allelism, *Bacillus thuringiensis*, cotton bollworm, F_1_ screen, genetically engineered crop, resistance management, second‐site noncomplementation, transgenic cotton

## Abstract

Evolution of resistance by insect pests reduces the benefits of extensively cultivated transgenic crops that produce insecticidal proteins from *Bacillus thuringiensis* (Bt). Previous work showed that resistance to Bt toxin Cry1Ac, which is produced by transgenic cotton, can be conferred by mutations disrupting a cadherin protein that binds this Bt toxin in the larval midgut. However, the potential for epistatic interactions between the cadherin gene and other genes has received little attention. Here, we report evidence of epistasis conferring resistance to Cry1Ac in the cotton bollworm, *Helicoverpa armigera*, one of the world's most devastating crop pests. Resistance to Cry1Ac in strain LF256 originated from a field‐captured male and was autosomal, recessive, and 220‐fold relative to susceptible strain SCD. We conducted complementation tests for allelism by crossing LF256 with a strain in which resistance to Cry1Ac is conferred by a recessive allele at the cadherin locus *HaCad*. The resulting F_1_ offspring were resistant, suggesting that resistance to Cry1Ac in LF256 is also conferred by resistance alleles at this locus. However, the *HaCad* amino acid sequence in LF256 lacked insertions and deletions, and did not differ consistently between LF256 and a susceptible strain. In addition, most of the cadherin alleles in LF256 were not derived from the field‐captured male. Moreover, Cry1Ac resistance was not genetically linked with the *HaCad* locus in LF256. Furthermore, LF256 and the susceptible strain were similar in levels of *HaCad* transcript, cadherin protein, and binding of Cry1Ac to cadherin. Overall, the results imply that epistasis between *HaCad* and an unknown second locus in LF256 yielded the observed resistance in the F_1_ progeny from the complementation test. The observed epistasis has important implications for interpreting results of the F_1_ screen used widely to monitor and analyze resistance, as well as the potential to accelerate evolution of resistance.

## 1 INTRODUCTION

Cotton, corn, and soybean have been genetically engineered to produce insecticidal proteins from *Bacillus thuringiensis* (Bt) that kill some key insect pests (Bravo, Likitvivatanavong, Gill, & Soberón, 2011; James, 2016; Sanahuja, Banakar, Twyman, Capell, & Christou, 2011). Farmers have planted these transgenic Bt crops on a cumulative total of over 830 million hectares since 1996 (James, 2016). The benefits of Bt crops include pest suppression, reduced reliance on conventional insecticides, and increased profits for farmers (Downes, Walsh, & Tay, 2016; Hutchison et al., 2010; Lu, Wu, Jiang, Guo, & Desneux, 2012; National Academies, 2016; Tabashnik et al., 2010; Wu, Lu, Feng, Jiang, & Zhao, 2008). However, increasingly rapid evolution of pest resistance to Bt crops is diminishing these benefits (Dively, Venugopal, & Finkenbinder, 2016; Tabashnik, 2016; Tabashnik, Brévault, & Carrière, 2013; Tabashnik & Carrière, 2017).

To help address this problem, the complementation test for allelism has been a key tool for monitoring resistance to Bt crops, isolating resistant strains from field populations, and understanding the genetic basis of resistance (Gould et al., 1997; Mahon, Downes, James, & Parker, 2010; Tabashnik, Liu, Finson, Masson, & Heckel, 1997; Wu, 2014; Zhang, Tian, et al., 2012). This test entails mating individuals homozygous for recessive resistance from different strains or populations and testing their F_1_ progeny for resistance. If the recessive alleles for resistance occur at one locus in one parent and a different locus in the other parent, the F_1_ progeny will be heterozygous for resistance at both loci. In this case, assuming no epistatic interactions between the two loci, the progeny are expected to be susceptible because of “allelic complementation” in which the dominant allele for susceptibility at each locus “complements” the recessive allele for resistance at each locus and restores the wild‐type phenotype. Conversely, if the recessive resistance alleles occur at the same locus in both parents, complementation does not occur; the progeny are resistant because they inherit two resistance alleles at the same locus.

The standard interpretation of the complementation test described above has been applied widely for estimating the frequency of resistance alleles and for starting resistant strains via the F_1_ screen, where field‐collected insects are mated individually to insects from a laboratory strain that is homozygous for recessive resistance (Downes et al., 2016; Gould et al., 1997; Huang et al., 2012; Liu et al., 2008; Siegfried et al., 2014; Wenes et al., 2006; Yue et al., 2008; Zhang, Tian, et al., 2012). It also has been used extensively for comparing the genetic basis of resistance between strains (Baxter et al., 2005; Camargo, Castañera, Farinós, & Huang, 2017; Fabrick & Tabashnik, 2012; Farias et al., 2014; Mahon, Downes, & James, 2012; Mahon, Olsen, & Downes, 2008; Mahon et al., 2010; Tabashnik, Gould, & Carrière, 2004; Tabashnik et al., 1997; Tabashnik, Liu, et al., 2004; Wang et al., 2017; Wirth, Walton, & Federici, 2010). However, interpretation of this test can be complicated by a type of epistasis called second‐site noncomplementation (SSNC), which is also referred to as nonallelic noncomplementation (Hawley & Gilliland, 2006). SSNC occurs when epistatic interactions between loci block restoration of the wild‐type phenotype in the F_1_ progeny, even though the recessive mutations occur at different loci (Hawley & Gilliland, 2006). The term “second‐site” refers to a second locus, and “noncomplementation” refers to failure to restore the wild‐type phenotype. Mutations at a second locus can cause SSNC via physical interactions between the proteins encoded by the two loci or by regulatory effects on expression of the first locus (Hawley & Gilliland, 2006). Although SSNC has been known in model organisms and humans for decades (Kajiwara, Berson, & Dryja, 1994; Stearns & Botstein, 1988), it has received little or no previous attention in the resistance management literature.

Here, we report that SSNC can affect resistance to Bt toxin Cry1Ac in the cotton bollworm, *Helicoverpa armigera* (Hübner), one of the world's most damaging pests of cotton and other crops (Anderson, Tay, McGaughran, Gordon, & Walsh, 2016; Czepak, Albernaz, Vivan, Guimaräes, & Carvalhais, 2013). In China, transgenic cotton producing Bt toxin Cry1Ac has been planted since 1997, which has reduced insecticide use against this pest and enhanced biological control by natural enemies, thereby providing substantial economic, environmental, and social benefits (Lu et al., 2012; Wu et al., 2008).

Although extensive planting of Bt cotton in northern China has increased the frequency of resistance in *H. armigera*, no major field failures have been reported (Jin et al., 2015; Liu et al., 2010; Zhang, Tian, et al., 2012; Zhang et al., 2011). Previous work has demonstrated the diverse genetic basis of resistance of Cry1Ac in *H. armigera*, including mutations affecting midgut proteins such as the transporter protein ABCC2, an aminopeptidase N, and the cadherin protein that binds Cry1Ac (Liu et al., 2014; Wu, 2014; Xiao et al., 2014, 2016, 2017; Xu, Yu, & Wu, 2005; Yang, Chen, Wu, Yang, & Wu, 2007; Yang, Chen, Wu, Yang, et al., 2006; Zhang, Wu, Yang, Tabashnik, & Wu, 2012; Zhang et al., 2009; Zhao, Jin, Yang, & Wu, 2010).

Here, we used the F_1_ screen to initiate a novel Cry1Ac‐resistant strain (LF256). First, a field‐captured male from Langfang, China, was mated to a female from the resistant SCD‐r1 strain that is homozygous for the recessive resistance allele r_1_ at the cadherin locus (*HaCad)* (Yang et al., 2009). Based on the lack of complementation in the progeny from the F_1_ screen, we hypothesized that the resistance in the field‐captured male and in LF256 was based on alleles at *HaCad*. However, subsequent sequencing of DNA, as well as analyses of genetic linkage, transcription, expression, and binding, refuted that hypothesis. Thus, we conclude that resistance to Cry1Ac in the initial F_1_ screen reflects epistasis between *HaCad* and an independently segregating locus.

## 2 MATERIALS AND METHODS

### 2.1 Insects

The susceptible SCD strain of *H. armigera* was started with insects from the Ivory Coast, Africa, over 30 years ago and has been maintained in the laboratory without exposure to insecticides or Bt toxins (Yang et al., 2009). The resistant strain SCD‐r1 was established by introgression of the r_1_ allele of *HaCad* from the Cry1Ac‐resistant GYBT strain into the SCD strain and has shown 440‐ to 540‐fold resistance to Cry1Ac relative to SCD (Yang et al., 2009; Zhang, Tian, et al., 2012; Zhang, Wu, et al., 2012). Larvae were reared on an artificial diet, and adults were maintained as described previously (Zhang et al., 2011).

### 2.2 Origin of resistant strain LF256: field collection of moths and F_1_ screen

Moths were collected using light traps in cotton fields during June 2009 from Langfang in the Hebei Province of northern China. Each field‐derived moth was paired individually with a homozygous resistant moth (r_1_r_1_) of the opposite sex from the SCD‐r1 strain. From each of 128 resulting single‐pair families, 48 of the F_1_ offspring were tested in bioassays (see below) at a diagnostic concentration of activated toxin (1 μg Cry1Ac per cm^2^ diet) that previously killed 100% of larvae from susceptible strains and F_1_ larvae from SCD‐r1 X SCD, but only 4%–8% of SCD‐r1 (Jin et al., 2015; Zhang, Tian, et al., 2012; Zhang, Wu, et al., 2012; Zhang et al., 2011).

As detailed in the Results, resistant strain LF256 was isolated from the F_1_ offspring produced by a cross between field‐captured male #256 from Langfang and a female from SCD‐r1. LF256 was maintained in the laboratory and occasionally selected with 1 μg Cry1Ac per cm^2^ diet to kill individuals that were not resistant.

### 2.3 Toxins

Cry1Aa‐, Cry1Ab‐, and Cry1Ac‐activated toxins were purchased from Dr. Marianne P. Carey (Case Western Research University, USA). Cry2Ab protoxin was provided by the Institute of Plant Protection, Chinese Academy of Agricultural Sciences (CAAS), China. Cry1A toxins and Cry2Ab protoxin were produced according to Monnerat et al. (1999) and Wei et al. (2015), respectively.

### 2.4 Bioassays

We tested larvae individually in diet overlay bioassays with Cry1A toxins and Cry2Ab using previously described standard methods. For Cry1A toxins, we tested second instars that were starved for 4 hr and recorded mortality at 5 days (Zhang et al., 2011). For Cry2Ab, we tested neonates (24 hr old) and recorded mortality after 7 days, which is the method established in Australia for testing Cry2Ab against *H. armigera* (Mahon, Olsen, Garsia, & Young, 2007). In all bioassays, larvae were recorded as survivors if they were alive and weighed >5 mg at the end of the bioassay (Jin et al., 2013).

We used a series of five to seven concentrations of each toxin (including a control with no toxin) to estimate the concentration of Cry1Aa, Cry1Ab, Cry1Ac, and Cry2Ab that kills 50% of the larvae (LC_50_) for SCD and the resistant strain LF256. For each concentration, we tested 48 larvae from each strain against each toxin.

### 2.5 Mode of inheritance

To evaluate dominance and sex linkage, we used bioassays to determine responses to Cry1Ac of SCD, LF256, and their F_1_ progeny from reciprocal mass crosses between the susceptible strain SCD and the resistant strains LF256. For each cross, we put 30 males of one strain and 30 females of the other strain in one cage. We used survival at the diagnostic concentration to calculate the dominance parameter *h*, which varies from 0 for completely recessive resistance to 1 for completely dominant resistance (Liu & Tabashnik, 1997).

### 2.6 Interstrain complementation test for allelism

Allelism between the cadherin resistance allele in strain SCD‐r1 and resistance allele in strain LF256 was determined by the survival at the diagnostic concentration of Cry1Ac of two resistant strains (LF256 and SCD‐r1), a susceptible strain (SCD) and progeny from reciprocal crosses between each resistant strain and the susceptible strain SCD or the resistant strain SCD‐r1. For each mass cross, 30 males and 30 females were placed in one cage. At the same time, we set up single‐pair reciprocal crossed between LF256 and SCD‐r1 according to the method described by Jin et al. (2013). Ninety‐six second‐instar larvae of F_1_ progeny from each of ten single‐pair crosses (six from LF256 male X SCD‐r1, four from LF256 female × SCD‐r1) were tested with 1 μg/cm^2^ Cry1Ac. To quantify the results of the complementation tests, we calculated the index of commonality (*C*), which measures the extent to which resistance alleles in two resistant strains are expected to share a common locus (Zhang, Tian, et al., 2012). Values of *C* close to 0 indicate the resistance alleles in the two strains do not share a common locus, and values close to 1 indicate the resistance alleles in the two strains are expected to share a common locus (Zhang, Tian, et al., 2012).

### 2.7 Sequencing of cadherin cDNA

For the sequencing of the cDNA of cadherin, total RNA of midgut tissue from fifth instars was individually extracted for each strain using the SV total RNA isolation system (Promega, Madison, WI, USA) according to the manufacturer's instructions and reverse transcribed with the Moloney murine leukemia virus reverse transcriptase (Promega). Specific primers and TaKaRa *Premix Taq*™ (Shiga, Japan) were used to amplify four overlapping gene fragments (Table S1), and one clone was sequenced for each fragment. PCR products of the expected size were purified using the Wizard DNA purification system (Promega) and cloned into the pGEM‐T easy vector system (Promega). All clones were sequenced by Invitrogen (Shanghai, China). We obtained one full cDNA sequence from each of the 12 larvae, six from SCD and six from LF256.

### 2.8 qRT‐PCR assays

Total RNA isolation and synthesis of cDNA was performed as described above. Each sample used for qRT‐PCR analysis was pooled from five midguts from fifth instars, and six samples were prepared for each strain. Real‐time PCR samples were prepared in SYBR^®^ *Premix Ex Taq*™ (Tli RNaseH Plus; TaKaRa), and reactions were carried out using the 7500 RT‐PCR detection system (ABI, USA). qRT‐PCR included an initial incubation of 30 s at 95°C followed by 40 cycles of amplification at 95°C for 5 s, 60°C for 34 s. Relative transcript levels of target genes in the LF256 larvae compared with the control SCD strain were calculated using the 2^−ΔΔCT^ method. The reference gene *EF‐1*α, validated by and Yang, Chen, Wu, Yue, & Wu. (2006), was used for normalization. Primers and PCR conditions were optimized to reduce nonspecific amplification. PCR efficiency was similar for the target gene (96.1%) and the reference gene (98.8%). Table S1 provides the primer sequences.

### 2.9 Midgut brush border membranes (BBMV) preparation

BBMV were prepared from insect midguts by the differential magnesium precipitation method (Wolfersberger et al., 1987). Briefly, 10 midguts were dissected from fifth‐instar larvae of *H. armigera*, washed in ice‐cold 0.15 M NaCl solution, and then homogenized in 3 ml of MET buffer (pH 7.5, containing 300 mM mannitol, 5 mM EGTA, and 17 mM Tris–HCl). 3.5 ml of 24 mM MgCl_2_ was added and incubated on ice for 15 min. The homogenate was then centrifuged at 2,500 *g* for 10 min; the supernatant was transferred into a new tube and centrifuged at 30,000 *g* for 30 min; and the pellets were collected and dissolved in 800 μl of 10 mM Hepes buffer (pH 7.5, containing 130 mM KCl, and 10% glycerol). Prepared BBMVs were kept at −80°C until used. The protein concentration was determined by Bradford method with BSA as a standard.

### 2.10 Western blot and ligand blot analysis

For Western blot assays, the BBMV proteins (10 μg) were separated on SDS–PAGE (10%) and transferred to polyvinylidene difluoride (PVDF) membrane (Bio‐Rad, Hercules, CA, USA). The membrane was blocked with 2.5% (w/v) BSA in TBST (pH 7.5, 25 mM Tris, 3 mM KCl, 135 mM NaCl, 0.1% Tween‐20) for 1.5 hr and then washed three times with TBST. After blocking, all filter incubations and washes were performed in TBST. Proteins were detected with polyclonal anticadherin antibody (1/5,000; 1 hr) and a goat anti‐rabbit secondary antibody coupled with horseradish peroxidase (1/10,000; 1 hr) (Abgent, San Diego, CA, USA), followed by Super Signal chemiluminescence substrate (Thermo, Waltham, MA, USA) as indicated by the manufacturer.

For ligand blot analysis, BBMV proteins (10 μg) were also separated on SDS–PAGE (10%) and transferred to polyvinylidene difluoride (PVDF) membrane (Bio‐Rad). The membrane was blocked with 2.5% (w/v) BSA in TBST for 3 hr. The blocked membrane was incubated with 1 nM Cry1Ac toxin overnight at 4°C. After washing three times with 0.1% (w/v) BSA in TBST, proteins with bound Cry1Ac were detected with polyclonal anti‐Cry1Ac antibody (1/5,000; 1 hr) and a goat anti‐rabbit secondary antibody coupled with horseradish peroxidase (1/10,000; 1 hr) (Abgent, San Diego, CA, USA), followed by Super Signal chemiluminescence substrate (Thermo, Waltham, MA, USA) as indicated by the manufacturer.

Both the anti‐Cry1Ac and anticadherin antibodies were produced in New Zealand white rabbits that were immunized subcutaneously by Abgent (Suzhou, China). A synthetic HaCad peptide (HGMFEFEVEATDSRR) found in the 11th cadherin repeat (CR11) was synthesized, conjugated to keyhole limpet hemocyanin (KLH) carrier protein, and used as the cadherin antigen by Abgent. In the first week, 200 μg of the purified polypeptides was homogenized with 1 ml of complete Freund's adjuvant and then injected hypodermically into the back of the rabbit. The second injection was performed 3 weeks later, 200 μg of polypeptide was injected into the muscle of the rabbit's back. After 1 week, 100 μg of polypeptide with incomplete Freund′s adjuvant was injected in the same location for intensification. The specificity and titer of the antiserums were examined by Western blot.

### 2.11 Test for genetic linkage between resistance to Cry1Ac and the cadherin locus in LF256

In preparation for genetic linkage analysis, we used bioassays with LF256 and the F_1_ progeny of LF256 × SCD to identify concentrations of Cry1Ac that enabled identification of live resistant and susceptible larvae. Using the bioassay method described above, second instars were exposed for 5 days to diet with either 0.5 or 5 μg Cry1Ac per cm^2^ diet. We tested 96 larvae from LF256 and F_1_ at each concentration (total *n* = 384). At the higher concentration, the percentage of larvae that survived and weighed >5 mg was 56% for LF256 and 0% for F_1_, indicating that survivors weighing >5 mg at the higher concentration could be characterized as resistant. At the lower concentration, the percentage of larvae surviving and weighing <5 mg was 0% for LF256 and 90% for F_1_, indicating that larvae surviving and weighing <5 mg after exposure to the lower concentration could be characterized as susceptible.

To test for genetic linkage in LF256 between resistance to Cry1Ac and the cadherin locus, we made a single‐pair cross between a male from the LF256 strain (putative cadherin genotype r_x_r_x_) and a female from the SCD strain (ss). One male from the resulting F_1_ progeny (putative r_x_s) was backcrossed with a female from LF256 (putative r_x_r_x_), which is expected to yield backcross progeny with r_x_r_x_ and r_x_s in a 1:1 ratio. Using the bioassay method described above, second instars from the backcross progeny were exposed for 5 days to diet with either 0.5 or 5 μg Cry1Ac per cm^2^ diet. Based on responses in the preliminary bioassays summarized above, survivors at the higher concentration were scored as resistant and larvae that survived but weighed <5 mg at the lower concentration were scored as susceptible. The larvae scored as either resistant or susceptible were transferred to untreated artificial diet and reared for DNA extraction until they became fourth or fifth instars.

We predicted that if resistance to Cry1Ac in LF256 is linked with the cadherin locus, then in the bioassays of backcross progeny, only r_x_s would weigh <5 mg at the lower concentration (susceptible) and only r_x_r_x_ would survive at the higher concentration (resistant). To test these predictions, we identified the cadherin alleles in the parents (male from LF256 and female from SCD), in the F_1_ male and the female he mated with (Figure S2), and in 60 backcross progeny. From the backcross progeny, we identified the cadherin alleles in 30 larvae that survived but weighed <5 mg from the lower concentration (susceptible, expected to be 100% r_x_s) and in 30 larvae that survived and weighed >5 mg from the higher concentration (resistant, expected to be 100% r_x_r_x_). From each of the 64 individuals listed above, we isolated genomic DNA with phenol‐chloroform extraction, amplified PCR fragments using specific primers (Table S1), and sent the PCR products for sequencing to Invitrogen (Shanghai, China).

We used a single nucleotide polymorphism (SNP) in intron 8 of *HaCad* as a genetic marker to distinguish between alleles from SCD and LF256 in the linkage analysis (Figure S2). At this site, the LF256 male in the parental cross (F_0_) and the L256 female used to make the backcross were both homozygous for thymine (T), whereas the SCD female in the parental cross was homozygous for adenine (A). As expected, the F_1_ male that mated with the LF256 female to generate backcross progeny was heterozygous (AT) at this site (Figure S2).

## 3 RESULTS

### 3.1 Isolation of resistant strain of LF256

**Figure 1:**
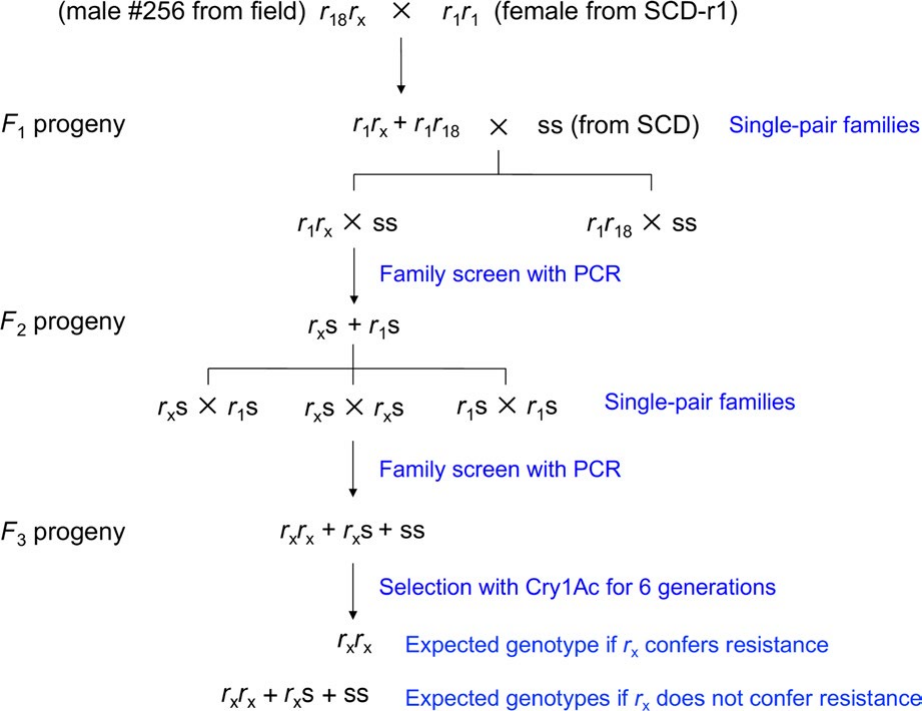
Establishment of the resistant LF256 strain, which was started by crossing male #256 captured from Langfang, China, with a female from the resistant SCD‐r1 strain. Cadherin genotypes are shown, with r and s indicating resistant and susceptible alleles, respectively. Alleles r_1_ and r_18_ each have a premature stop codon. Based on the initial complementation test results, the second cadherin allele in the field‐captured male was tentatively named r_x_ (as shown above), to indicate its hypothesized role in resistance. However, analysis of resistant strain LF256 revealed that resistance in this strain was not conferred by alleles at the cadherin locus (see Section 2.1). In particular, r_x_ was not fixed in the LF256 strain, which refutes the hypothesized role of this cadherin allele in resistance

As part of an F_1_ screen, we paired a field‐captured male (#256) from Langfang with a female from the resistant strain SCD‐r1, which was homozygous for the cadherin resistance allele (r_1_r_1_; Figure 1). Survival of their F_1_ offspring at the diagnostic concentration (1 μg Cry1Ac per cm^2^ diet) was 85.4% (*n* = 48), suggesting the male had two resistance alleles at the cadherin locus or that he was homozygous for dominant resistance at another locus. To evaluate these hypotheses, we conducted a series of experiments, including isolation of resistant strain LF256. Sequencing of the cDNA in the F_1_ survivors revealed one new cadherin allele from the field‐captured male with a 12‐bp deletion at 2,403 bp and insertions of 4 and 7 bp at 4,247 and 4,257 bp of the cDNA sequence, respectively, yielding a premature stop codon in the region coding for the eleventh cadherin repeat (CR11) of the ectodomain (Figure S1). Here, we name this mutant cadherin allele r_18._ The other cadherin allele from the field‐captured male lacks insertions and deletions (indels) and has 98.4% amino acid identity with a wild‐type cadherin allele from SCD (GenBank accession no. AFB74168.1). Nonetheless, to represent its hypothesized role in resistance, we tentatively named it r_x_.

To establish the LF256 strain (Figure 1), we conducted 30 single‐pair crosses between the F_1_ survivors and the susceptible SCD strain. Using PCR with primers specific for r_18_ (Table S1), we identified the parents of 12 of the 20 productive single‐pair crosses that had r_18_ and discarded their offspring. We retained the offspring (F_2_ progeny) from the eight other families, which were produced by single‐pair crosses between r_1_r_x_ and ss. Next, we made 100 single‐pair crosses among the F_2_ progeny. Using PCR with primers specific for r_1_ (Table S1), we identified the parents of 45 of the 55 productive single‐pair crosses that had r_1_ and discarded their offspring. We pooled the offspring (F_3_ progeny) from the other 10 families, which were produced by crosses between r_x_s and r_x_s. In the subsequent generations, we allowed mass mating, selected larvae at increasing concentrations of Cry1Ac (1–5 μg Cry1Ac per cm^2^ diet), and reared the survivors to continue the strain. After six successive generations of selection, LF256 had 220‐fold resistance to Cry1Ac relative to the susceptible SCD strain (Table 1).

**Table 1:** Resistance to Cry1Ac and cross‐resistance to Cry1Aa, Cry1Ab, and Cry2Ab of LF256 relative to susceptible strain SCD of *Helicoverpa armigera*

Strain	Bt toxin	LC_50_ (95% FL)^a^	Slope ± *SE*	RR^b^
LF256	Cry1Ac	6.71 (4.56–11.3)	0.91 ± 0.16	224
	Cry1Ab	8.25 (5.85–13.1)	1.16 ± 0.18	34
	Cry1Aa	>20^c^	NA^d^	>67
	Cry2Ab	0.36 (0.29–0.46)	1.74 ± 0.19	1.5
SCD	Cry1Ac	0.03 (0.02–0.05)	1.12 ± 0.13	1.0
	Cry1Ab	0.24 (0.14–0.36)	1.39 ± 0.25	1.0
	Cry1Aa	0.30 (0.15–0.52)	0.80 ± 0.26	1.0
	Cry2Ab	0.23 (0.11–0.56)	1.72 ± 0.22	1.0

a Concentration (μg toxin per cm^2^ diet) killing 50% of larvae and its 95% fiducial limits, *n* = 336 larvae tested from each strain against each toxin.

b Resistance ratio = LC_50_ for a strain divided by the LC_50_ for the susceptible strain SCD.

c Mortality was 17% at 20 μg Cry1Aa per cm^2^ diet, the highest concentration tested.

d Not available.

### 3.2 Cross‐resistance of LF256

Relative to SCD, LF256 had >67‐fold cross‐resistance to Cry1Aa and 34‐fold cross‐resistance to Cry1Ab (Table 1). Although the LC_50_ of Cry2Ab was 1.5‐fold higher for LF256 than SCD, this difference was not statistically significant based on the conservative criterion of no overlap between the 95% fiducial limits (Table 1).

### 3.3 Mode of inheritance of resistance in LF256

For the F_1_ progeny from crosses between LF256 and susceptible strain SCD, survival at the diagnostic concentration did not differ significantly between the two reciprocal crosses (2.1% for progeny from female LF256 X male SCD, and 6.2% for female SCD X male LF256, *n* = 96 for each cross, Fisher's exact test, *p* = .28). These results demonstrate that effects of maternal inheritance and sex linkage were not evident, indicating autosomal inheritance of resistance.

**Figure 2:**
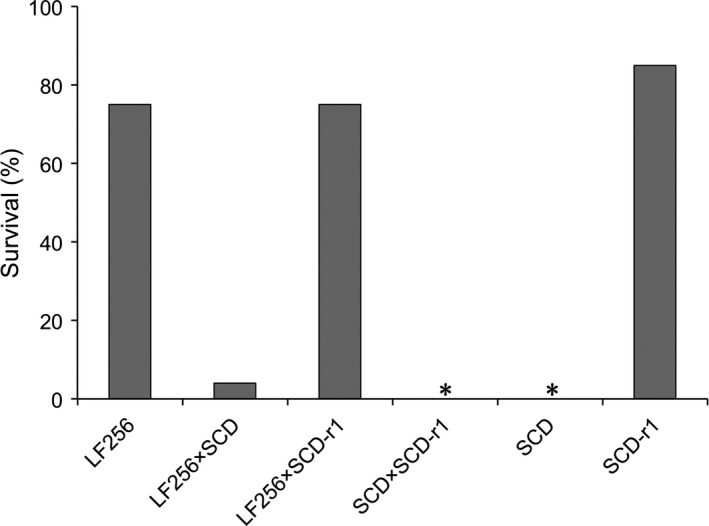
Survival at the diagnostic concentration of Cry1Ac of two resistant strains (LF256 and SCD‐r1), a susceptible strain (SCD) and the progeny from crosses between strains. Asterisks indicate 0% survival for the SCD strain and the F_1_ progeny from the cross between the SCD strain and the SCD‐r1 strain

The value for dominance (*h*) calculated from the pooled survival of the F_1_ progeny at the diagnostic concentration (4.2%, Figure 2) was 0.06, indicating almost completely recessive inheritance of resistance. These results refute the hypothesis that field‐captured male #256, the source of the resistance alleles in strain LF256, was homozygous for dominant resistance. To test the alternative hypothesis that he had two resistance alleles at the cadherin locus, we conducted a complementation test for allelism as described below.

### 3.4 Complementation test for allelism between LF256 and SCD‐r1

Survival at the diagnostic concentration was 75% (72/96) for LF256, 85% (82/96) for SCD‐r1, and 75% (144/192) for the F_1_ progeny from a mass cross between LF256 and SCD‐r1 (Figure 2). Consistent with the results from the mass cross, mean survival of F_1_ progeny from 10 single‐pair crosses between LF256 and SCD‐r1 was 75% (range: 71%–80%). The similar survival of the F_1_ progeny compared to the resistant parent strains indicates lack of complementation and yields a value of 0.91 for the commonality index (*C*), which suggests resistance in LF256 and SCD‐r1 is conferred by alleles at the same locus, that is, the cadherin locus.

### 3.5 Cadherin cDNA sequence

**Figure 3:**
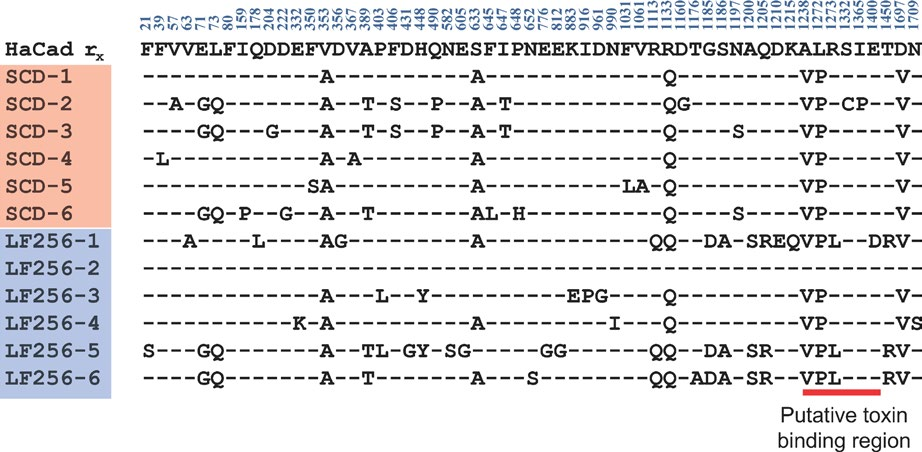
Alignment of polymorphic amino acids predicted from sequencing the cadherin gene in male #256, resistant strain LF256, and susceptible strain SCD of *Helicoverpa armigera*. Dashes indicate the amino acids are the same as in the r_x_ allele from male #256. Only LF256‐2 was identical to r_x_. The red line shows the putative toxin‐binding region of HaCad. No mutations were found in SCD or LF256 in amino acids 1,422–1,440, which are especially important in binding (Zhang et al., 2017)

Based on sequencing of cadherin cDNA, no indels occurred in LF256, the predicted cadherin amino acid sequence varied within the LF256 and SCD strains, and no consistent differences occurred between strains (Figure 3). The absence of indels in LF256 and the lack of consistent differences between strains suggest that variation in cadherin amino sequence does not cause resistance to Cry1Ac in LF256. Furthermore, if resistance of LF256 to Cry1Ac was conferred by or tightly genetically linked with the cadherin locus, then all individuals in LF256 would carry the r_x_ allele inherited from male #256 (Figure 1). However, the frequency of r_x_ in LF256 was only 0.16 (1 of 6; Figure 3), which differs significantly from the expected frequency of 1.0 if resistance was conferred by or genetically linked with the cadherin locus (Fisher's exact test, *p* = .015). The observed frequency of 0.16 does not differ significantly from the expected frequency of 0.5 if resistance was not genetically linked with the cadherin locus (Fisher's exact test, *p* = .55).

### 3.6 Resistance in LF256 is not genetically linked with the cadherin locus

Analysis of 60 larvae from a backcross family produced by crossing an F_1_ male (from LF256 X SCD) with an LF256 female confirmed that resistance to Cry1Ac was not genetically linked with cadherin in LF256. For 30 of the backcross larvae that survived when exposed to diet treated with 5 μg Cry1Ac per cm^2^ diet and thus were scored as resistant, the observed ratio of cadherin genotypes was 17 r_x_r_x_ to 13 r_x_s. This observed outcome differs significantly from the outcome of 100% r_x_r_x_ survivors predicted under the hypothesis of tight linkage between cadherin and resistance (Fisher's exact test, *p* < .0001). For 30 of the backcross larvae that survived but weighed <5 mg after exposure to diet with 0.5 μg Cry1Ac per cm^2^ diet and thus were scored as susceptible, the observed ratio of cadherin genotypes was 13 r_x_r_x_ to 17 r_x_s. This observed outcome also differs significantly from the outcome of 100% of susceptible individuals with the genotype r_x_s predicted under the hypothesis of tight linkage between cadherin and resistance (Fisher's exact test, *p* < .0001). In addition, the observed ratio of r_x_r_x_:r_x_s did not differ significantly from the 1:1 ratio expected under random segregation (i.e., no linkage with cadherin) for either group of 30 larvae (Fisher's exact test, *p* = .80 in each case). Moreover, despite the major difference in resistance phenotype between the two groups of 30 larvae, the observed ratio of cadherin genotypes did not differ significantly between them (Fisher's exact test, *p* = .44), which supports the conclusion that resistance in LF256 was not linked with the cadherin locus.

### 3.7 Cadherin transcript and protein levels, and binding of Cry1Ac to cadherin in LF256

Analysis of cadherin RNA by qRT‐PCR revealed no significant difference between LF256 (mean = 1.07, *SE* = 0.08) and SCD (mean = 1.0, *SE* = 0.05; *t* test, *t* = 2.23, *df* = 4, *p* = .52).

**Figure 4:**
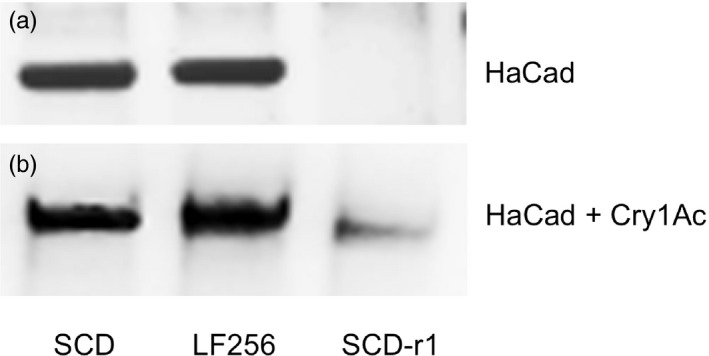
Analysis of the SCD (susceptible), LF256 (resistant), and SCD‐r1 (resistant) strains of *Helicoverpa armigera*. (a) Western blot of cadherin protein. (b) Ligand blot of binding of Cry1Ac to cadherin protein. Both blots show similar bands for LF256 and SCD, and no band (a) or a weaker band (b) for SCD‐r1

In Western blots, the band for cadherin was similar in LF256 and SCD (Figure 4a). Consistent with previous work (Wang et al., 2016), the band for cadherin was absent in SCD‐r1 (Figure 4a). In parallel with the Western blot results, ligand blots showed binding of Cry1Ac to cadherin was similar in LF256 and SCD, but weaker in SCD‐r1 (Figure 4b).

## 4 DISCUSSION

When we conducted an F_1_ screen, a method used widely to monitor resistance to Bt crops, a cross between a field‐captured male (#256) of *H. armigera* and a female from the SCD‐r1 strain homozygous for a recessive cadherin resistance allele yielded 85.4% resistant offspring. The standard interpretation of the results from this complementation test is that the male carried two resistance alleles at the cadherin locus (*HaCad*). However, sequencing of cDNA revealed that while one of the cadherin alleles (r_18_) from this male harbors a premature stop codon expected to confer resistance, the second allele (r_x_) lacks deletions, insertions, or stop codons and encodes an amino acid sequence 98.4% identical to that of the susceptible strain SCD (Figure S1).

To investigate these paradoxical results, we incorporated the second allele from male #256 into a new resistant strain (LF256) using single‐pair crosses with susceptible strain SCD followed by family screening with PCR and selection with Cry1Ac (Figure 1). This yielded 220‐fold autosomal recessive resistance to Cry1Ac in LF256 relative to the susceptible strain (Table 1, Figure 2). In a second set of complementation tests, crosses between LF256 and resistant strain SCD‐r1 produced resistant F_1_ offspring. This outcome is consistent with the results of the initial F_1_ screen, implying again that the second cadherin allele (r_x_) from male #256 confers resistance to Cry1Ac. Nonetheless, the results from DNA sequencing and analyses of genetic linkage, as well as levels of transcript, protein, and binding (Figures S1 and 2, 3, 4), refute the hypothesis that resistance to Cry1Ac in LF256 is conferred by or even linked with the cadherin locus.

The simplest explanation for all of the data is that SSNC between the cadherin locus and an independently segregating locus conferred resistance in the F_1_ progeny from the cross between LF256 and SCD‐r1. An alternative hypothesis is that SSNC did not occur and SCD‐r1 was homozygous for resistance at two loci: the cadherin locus and an independently segregating locus that also harbored the alleles conferring resistance in LF256. However, this is unlikely because SCD‐r1 was created by introgressing the cadherin r1 allele into SCD using a series of crosses and family selection for the r1 allele by allele‐specific PCR (Yang et al., 2009). Thus, resistance alleles at loci unlinked with cadherin are expected to be rare or absent in SCD‐r1. Nonetheless, we cannot exclude the hypothesis of epistasis between the cadherin locus and two or more independently segregating loci (Ehrenreich, 2017). As far as we know, this study is the first to report evidence of SSNC affecting resistance to Bt toxins or other insecticides.

The mode of action of Bt toxins involves several insect midgut proteins encoded by different genes (Pardo‐Lopez, Soberon, & Bravo, 2013), which provides the opportunity for epistasis to occur via interactions among these proteins. Epistasis conferring resistance to Bt toxins could also occur via regulatory interactions between loci (Baxter et al., 2011; Guo et al., 2015; Tiewsiri & Wang, 2011). The results here imply that heterozygosity for resistance at both the cadherin locus and a second, unlinked locus are sufficient to disrupt the toxic pathway and thereby confer resistance, even though each of the two mutations alone is recessive. The putative locus other than cadherin conferring resistance to Cry1Ac in LF256 remains to be identified. Candidates include the previously identified genes other than *HaCad* that are associated with resistance to Cry1Ac in other strains of *H. armigera* (Chen et al., 2015; Wu, 2014). In particular, a premature stop codon in *HaABCC2* confers autosomal recessive resistance to Cry1Ac in another strain of *H. armigera* from Langfang (Xiao et al., 2014). Furthermore, ABCC2 and cadherin may interact synergistically to boost toxicity of Cry1A proteins in *Bombyx mori* and *Heliothis virescens* (Bretschneider, Heckel, & Pauchet, 2016; Tanaka, Endo, Adegawa, Kikuta, & Sato, 2016; Tanaka et al., 2013). However, results from a cross with *H. virescens* imply that individuals heterozygous for resistance at both the cadherin and ABCC2 loci were susceptible to Cry1Ac, which means that SSNC did not occur (Jurat‐Fuentes, Gould, & Adang, 2002). The resistant strain YHD2 was used in this cross when it showed no binding of Cry1Aa, Cry1Ab, and Cry1Ac (Jurat‐Fuentes et al., 2002), which was later interpreted to indicate homozygosity for resistance at both loci (Gahan, Pauchet, Vogel, & Heckel, 2010). Thus, the cross between YHD2 and susceptible strain YDK is expected to have produced F_1_ offspring heterozygous for resistance at the cadherin and ABCC2 loci. Because SSNC can vary among specific alleles (Hawley & Gilliland, 2006), this finding does not exclude the possibility of SSNC occurring between some of the cadherin and ABCC2 resistance alleles in *H. armigera*.

The results here have important implications for interpreting outcomes from the F_1_ screen for resistance and for evolution of resistance. As demonstrated here, resistance of F_1_ progeny produced by crosses between individuals from different strains or populations with recessive resistance can occur even when the resistance is not conferred by alleles at a shared locus. Therefore, the results from complementation tests may suggest the role of a particular locus in resistance, but they are not definitive. A more robust conclusion can be reached if the complementation results are augmented with evidence of genetic linkage between resistance and the putative resistance locus, disruptive mutations in the putative resistance locus, or both.

This new perspective applies to previously described strains of *H. armigera* from China where the only evidence that their resistance to Cry1Ac is conferred by cadherin alleles is resistance of F_1_ progeny in complementation tests involving crosses with strains homozygous for the cadherin allele r_1_ (SCD‐r1 and GYBT). Sequencing the putative cadherin resistance alleles in F_1_ survivors from complementation tests in three studies yielded eight distinct alleles (r_2_ to r_9_) other than r_1_ with disruptive mutations, but also 10 individuals with no major deletions, insertions, or premature stop codons in their putative cadherin resistance alleles (including r_10_ to r_14_; Yang et al., 2007; Zhang, Tian, et al., 2012; Zhao et al., 2010). Without further analyses, we cannot determine whether the resistance in the F_1_ survivors from these 10 cases was conferred by alleles at the cadherin locus or by SSNC as with LF256.

The potential for SSNC changes the interpretation of results from F_1_ screens in all studies where additional data are not available to either confirm or refute the hypothesis that the resistance in F_1_ progeny from complementation tests is conferred by alleles at a locus that is shared between the parental strains or populations. However, the results here do not alter the well‐established understanding that the F_1_ screen can detect recessive alleles at the same locus in both parents used in the cross, as well as nonrecessive alleles at any locus. The results here demonstrate that the F_1_ screen also can detect recessive alleles at any locus for which SSNC occurs between the resistance alleles in the parents used in the cross.

The type of SSNC discovered here also has the potential to accelerate evolution of resistance. Individuals heterozygous for resistance at both the cadherin locus and the second locus are resistant. Therefore, in effect, the second locus can be considered a modifier that increases the dominance of resistance conferred by the cadherin locus. Because resistance evolves faster as dominance increases (Tabashnik & Carrière, 2017; Tabashnik, Gould, et al., 2004), the epistasis between cadherin and the second locus is expected to hasten evolution of resistance. It remains to be determined how commonly SSNC occurs with resistance to Bt toxins in *H. armigera* and other insects.

## DATA ARCHIVING STATEMENT

Sequence data for two alleles of *HaCad* from this study have been deposited in the National Center for Biotechnology Information (GenBank No. MF375235 for the r_18_ allele and MF375236 for the r_x_ allele). Table S1 provides sequences of primers used in this study.
