# Supplementary material for: Epistasis confers resistance to Bt toxin Cry1Ac in the cotton bollworm
Source: Evol Appl. 2018 Feb 10;11(5):809–19. doi: 10.1111/eva.12598 (PMC5979638; doi:10.1111/eva.12598)
Supplement: Supplementary file 1 [file EVA-11-809-s001.docx]

**Supporting information**

**Table S1.** Nucleotide primers used to obtain cDNA fragments of cadherin and to perform qPCR.

| Fragment name | Forward sequence (5’-3’) | Reverse sequence (5’-3’) |
| --- | --- | --- |
| For amplifying *HaCad* cDNA fragments | | |
| Cadherin-1 | ATGGCAGTCGACGTGAGAATA | ctatgtagaacgcctcgtgag |
| Cadherin-2 | GATGTGGTCATCATCGTGAAC | cgtgttgaagtctatgtccac |
| Cadherin-3 | GCAGCCTCAGGAGTCGTTATA | gttgtaaggtctgatgaccag |
| Cadherin-4 | TCAACATGATCACCATAGAGAG | ttatcttctgaactgtgtgttcg |
|  |  |  |
| For specific detection of the r1 allele of *HaCad* | | |
| Cad-r1 | agacagggacactcttgagaag | cttcacacatgatgttcctcg |
|  |  |  |
| For specific detection of the r18 allele of *HaCad* | | |
| Cad-r18 | TATAATCAAGTGCGTCCCAGAC | cgtgttgaagtctatgtccact |
|  |  |  |
| For genetic linkage analysis | | |
| Cad-Int8 | AGACAGGGACACTCTTGAGAAG | CTATGTAGAACGCCTCGTGAG |
|  |  |  |
| For performing qRT-PCR | | |
| EF-1α | GACAAACGTACCATCGAGAAG | GATACCAGCCTCGAACTCAC |
| Cadherin-qPCR | ATGGAGGAAACTGCGATGAC | CCAACATGTGATGGTTCTGC |





**Figure S1** Amino acid sequences of *HaCad* from SCD, the *rx* and *r_18_* alleles deduced from cDNA. Identical residues are designated by dashes. The solid box shows the deletion of 4 Amino acid residues at CR6, and an asterisk shows the premature stop codon in *r_18_* at CR11. Horizontal arrows specify start sites of putative domains. SIG, signal peptide; CR, cadherin repeat; MPR, membrane-proximal region; TM, transmembrane domain; CYT, cytoplasmic domain.


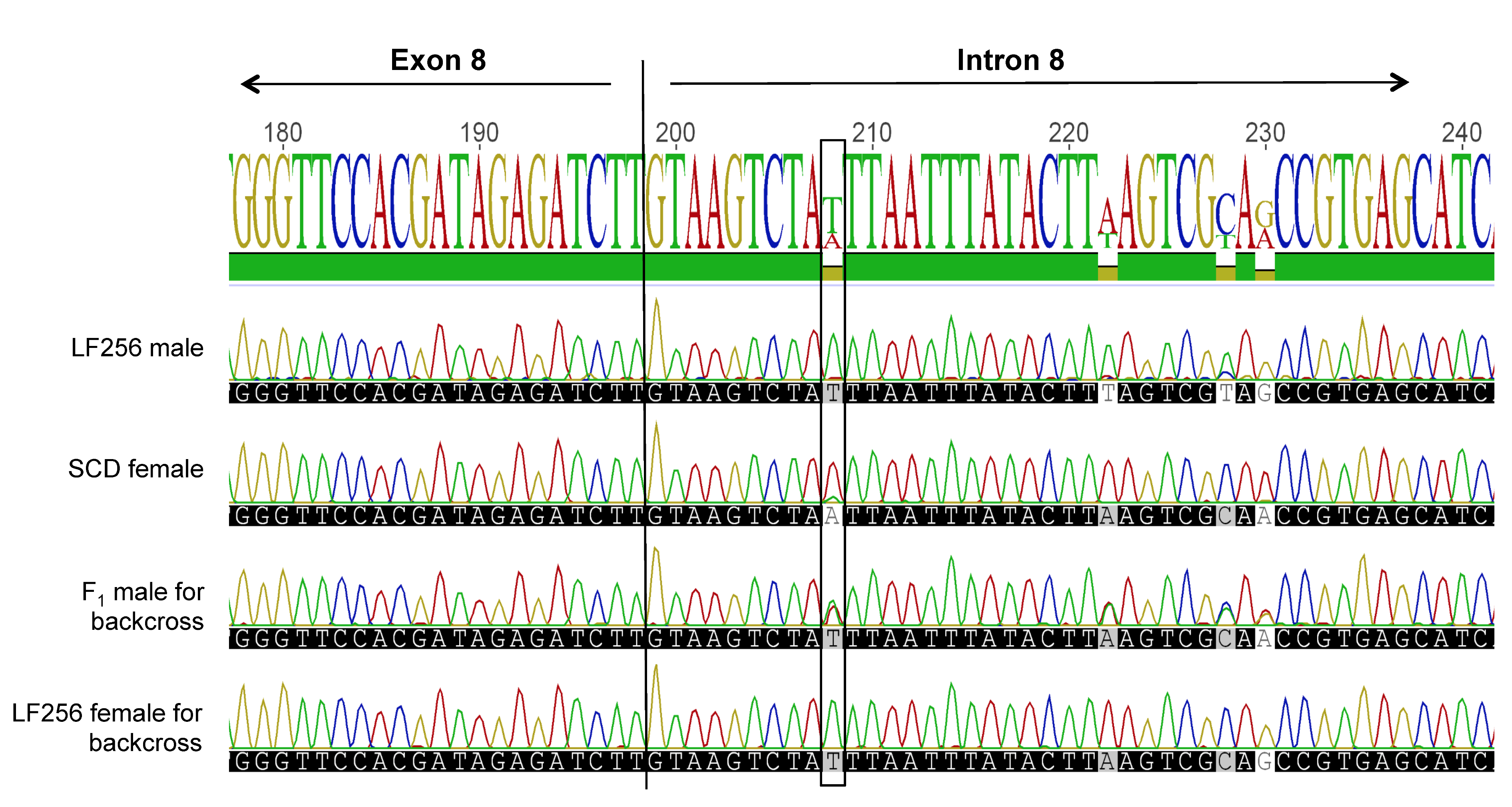


**Figure S2** The SNP marker (boxed) at intron 8 of *HaCad* used for the linkage analysis. A genomic DNA fragment (about 680 bp) containing the whole intron 8 of *HaCad* was amplified from individual samples with a pair of specific primers. Primer sequences were detailed in Table S1, and the forward primer was located in exon 8 and the reverse primer in exon 9. PCR products were directly sequenced with the forward primer used for DNA amplification. Within this DNA fragment, a single nucleotide polymorphism (SNP) in intron 8 of *HaCad* (boxed) was selected as a genetic marker for the linkage analysis. At this SNP site, the LF256 male and the SCD female (F_0_ parents of a single pair cross) were homozygous for thymine (T) and adenine (A) respectively. An F_1_ male (A/T at the SNP site) was backcrossed with an L256 female (TT at the SNP site).
